# What are the implications for patient safety and experience of a major healthcare IT breakdown? A qualitative study

**DOI:** 10.1177/20552076211010033

**Published:** 2021-04-19

**Authors:** Arabella Scantlebury, L Sheard, Cindy Fedell, J Wright

**Affiliations:** 1 York Trials Unit, Department of Health Sciences, University of York, York, England; 2 Bradford Teaching Hospitals NHS Foundation Trust, Bradford Royal Infirmary, Bradford, England

**Keywords:** Patient safety, patient experience, quality, qualitative, secondary care, NHS, crisis, technology

## Abstract

**Introduction:**

To explore the impact of a three-week downtime to an electronic pathology system on patient safety and experience.

**Methods:**

Qualitative study consisting of semi-structured interviews and a focus group at a large NHS teaching hospital in England. Participants included NHS staff (*n* = 16) who represented a variety of staff groups (doctors, nurses, healthcare assistants) and board members. Data were collected 2–5 months after the outage and were analysed thematically.

**Results:**

We present the implications which the IT breakdown had for both patient safety and patient experience. Whilst there was no actual recorded harm to patients during the crisis, there was strong and divided opinion regarding the potential for a major safety incident to have occurred. Formal guidance existed to assist staff to navigate the outage but there was predominantly a reliance on informal workarounds. Junior clinicians seemed to struggle without access to routine blood test results whilst senior clinicians seemed largely unperturbed. Patient experience was negatively affected due to the extensive wait time for manually processed diagnostic tests, increasing logistical problems for patients.

**Conclusion:**

The potential negative consequences on patient safety and experience relating to IT failures cannot be underestimated. To minimise risks during times of crisis, clear communication involving all relevant stakeholders, and guidance and management strategies that are agreed upon and communicated to all staff are recommended. To improve patient experience flexible approaches to patient management are suggested.

## Introduction

In 2016, a large NHS teaching hospital in England experienced a failure to its electronic pathology system. At the time of the outage, pathology services were provided by a neighbouring hospital. The outage affected the neighbouring hospital’s electronic laboratory information system and so electronically transferring results between the two organisations was not possible during the three-week outage. The disruption caused was unprecedented, partly, because of the outages duration, but also because it affected two large NHS teaching hospitals, which are reliant on pathology for a number of clinical services (e.g. transfusion, microbiology). The outage therefore not only placed strain across one organisation’s ability to provide clinical services, but an entire local health community.

Over the last two decades, there has been a global drive towards digitising healthcare.^1^ As a result, there are a number of key clinical areas for which electronic systems are integral to day-to-day patient management, diagnosis and decision-making. For example, pathology and Picture Archiving and Communication Systems (PACS).

As healthcare systems become more ‘digitised’, organisations and individuals are required to change how they work to integrate the use of technology and are becoming increasingly dependent on technology to deliver healthcare.^2^ However, no technology is 100% reliable, and so it is important to understand what happens when the technology that healthcare organisations, and professionals, have become so reliant on fails. More specifically, what are the implications of catastrophic IT failures to the safety of healthcare and patient experience?

Existing evidence on e-health has focussed on its implementation, and in particular, the potential for electronic systems to improve the quality and safety of healthcare.^1,3–18^ Much less attention, has been given, to the potential negative impacts of e-health,^19^ with the effects of catastrophic IT downtimes on the quality and safety of healthcare a particularly under-researched area.^2,20^ When considering the patient safety literature more broadly, there is a large international evidence base on emergency preparedness and response, however, few studies have explored the impact of major crises on healthcare.^21–24^ Likewise, UK policy has largely focussed on the potential benefits of digitising healthcare and technology implementation.^25–28^ In 2017, a worldwide cyber-attack caused disruption to over a third of NHS hospitals and resulted in an independent report on the attack’s impact being commissioned by the Department of Health.^29^ Since the attack, the digital resilience of NHS organisations has been high on the UK policy agenda and local organisations have been urged to improve their digital infrastructure and security.^29^

This paper does not aim to determine the technical reasons behind the IT failure at the teaching hospital in question, but aims to contribute to the limited evidence surrounding the impact of major catastrophic events on healthcare, by reporting on the impact of a major IT power outage – a three-week downtime to an electronic pathology system – on a single NHS organisation. Through interviews and focus groups, we captured the perspectives and experiences of board members and clinical staff and aim to obtain an in-depth understanding of the impact of the outage on patient safety and experience. In doing so, we aim to generate wider lessons that can be applied to organisations during other rare catastrophic events and promote sharing of lessons between organisations during times of crisis.

## Methods

### Study design

A qualitative exploratory study comprising semi-structured interviews and a focus group was adopted to explore an organisation’s response to a pathology system’s downtime at a NHS hospital. Data collection took place between November 2016 and February 2017, approximately 2–5 months after the pathology system’s downtime. The study was designed in response to a major healthcare crisis and so a flexible and pragmatic approach to recruitment and data collection was required. One of the challenges we faced was recruiting staff to interviews at a time when the hospital was experiencing unprecedented demand. To mitigate against this, we chose to purposively interview key informants who represented areas most affected by the outage and we were mindful not to interview more informants than necessary to reduce burden on key clinical roles. Therefore, our sample was limited to 16 key clinicians and hospital board members, who represented a range of staff groups, grades and specialties.

Research governance approval was obtained from the University of York Health Sciences Research Governance Committee on 25 October 2016.

### Setting and pathology system

The study was conducted at a large NHS foundation trust in the England, which provides hospital services to approximately 500,000 people and specialist services to 1.1 million people.

At the time of the power outage, the hospital’s pathology services were provided under contract with another local NHS hospital – the host organisation. The pathology service is an end-to-end service from sample analysis to the provision of an electronic result and covers all laboratory disciplines including: biochemisty, blood sciences, blood transfusion, cellular pathology, immunology and specialist tests. The system routinely processes an estimated 2000 samples per day, provides pathology services to over 60 General Practices and is a tertiary service for specialist tests to a wider population. To protect the anonymity of both organisations more specific details of the pathology system and dates for the outage are not provided.

### Sampling and recruitment

We present data from the perspective of those at a NHS organisation. Key members of the trust board were initially recruited as it was anticipated that they would provide a unique perspective on the organisation’s response to the outage. At the end of each interview, board members were asked to identify the clinical areas that they felt were most affected during the outage and provide the contact details of consultants in these areas. We acknowledge the limitations to this approach. However, this research was conducted in direct response to a major crisis and so this was considered the most efficient recruitment method. Within the wards that were identified, a purposive sampling frame was adopted to ensure that a range of staff groups and grades were recruited to understand their perceptions and experiences of the outage and its perceived impact.

Participants were recruited to interview via telephone and email. Significant difficulty was encountered in recruiting junior doctors to individual interviews and so a focus group was conducted for these participants. The focus group took place during the junior doctors’ lunch hour, with participants recruited via a junior doctors’ WhatsApp group.

### Participants

Sixteen participants consented and participated in the research. Seven junior doctors participated in a focus group and nine individual interviews were conducted with three members of the trust board, three consultants, one pathology consultant, one nurse and 1 Health Care Assistant/patient flow manager. Participants represented a range of wards including: oncology, cardiology, general surgery, Acute Medical Unit (AMU), elderly care, pathology and intensive care/anaesthesia.

### Data collection

The focus group and interviews were conducted face-to-face, were semi-structured and lasted between 17 and 57 min. Two topic guides were devised; one for interviews and one for the focus group. Topic guides were developed by the research team and piloted with the director of informatics at the study site (Appendix 1 and 2). During the interviews and focus group, participants were asked about the organisational and clinical response to the outage and its impact on the organisation, the local healthcare community and clinical practice. Interviews with members of the trust board were more focussed on the organisational response and the factors affecting this. Topic guides provided a framework for data collection, however a flexible approach to the format of topic guides and questions posed was adopted to ensure participants could discuss issues they felt were important. Written informed consent was taken from all participants prior to each interview.

### Analysis

Interviews were audio-recorded and transcribed verbatim. Analysis was facilitated by use of the qualitative data management programme NVIVO (version 11). AS conducted the analysis with regular discussions with LS to discuss theme and sub-theme development. To ensure that a systematic approach to analysis was adopted, data were analysed using a thematic approach, following the stages outlined by Braun et al. (2006): detailed familiarisation with the data; code and theme development and data reporting.^30^ Theme and sub-theme development was largely deductive based on a-priori themes relating to issues included in the topic guide. An iterative approach to theme and sub-development was then adopted to refine and re-develop themes and sub-themes.

## Results

First, we set the scene by reporting descriptively key information that gives context to the outage. This is necessary for readers to be able to understand the resultant implications for patient safety and experience. All the information we portray was uncovered from the qualitative fieldwork and could be considered a theme in its own right. For the sake of brevity and concentration on our main findings of safety and experience, we have condensed this material into snapshots that are pivotal to understanding the eye of the storm. These are presented in Table 1.

**Table 1.** Summary of the hospital’s initial response to the outage.

Response	Description
Underestimation of the outage’s significance	The outage’s significance was perceived to be underestimated by the hospital both in terms of its expected duration and potential impact. Participants felt that there was a delayed response to the crisis, which had a ‘knock on effect’ on the hospital’s ability to manage the situation throughout the outage. The initial underestimation of the outage’s significance was perceived to be related to: communication issues between the hospital and the host organisation; the frequency of IT failures in the NHS and a perceived ‘weekend’ effect.
Crisis management plan	At the time of the outage, there was no agreed hospital-wide crisis management plan. Board members discussed how the organisation’s response was often iterative and reactive to the situation and events. Staff discussed the difficulties of trying to define a crisis management plan whilst doing the day job and how this was made more challenging by the organisation’s initial underestimation of the outage’s significance and subsequent delayed response.
Reverting to paper	When the outage occurred, the hospital reverted to a manual, paper-based pathology service, which struggled to cope with the increased volume of work this brought. This was attributed to the fact that pathology services have been reliant on IT systems for 30 years and also to the specialised nature of pathology which made it difficult to provide additional staff during the crisis. To give an estimate of scale, business as usual saw approximately 2,000 blood samples processed per day.
Identifying priority wards	To cope with the reduced processing capacity and additional demand of manual processing, wards and patients were identified which should be considered a priority. Critical areas were considered those where processing time was integral to patient safety, such as the Emergency Department(ED). Communication was issued to all clinical staff informing them that they should only be issuing requests to pathology for urgent or emergency cases.
Additional staff and resources	One of the consequences of reverting to a paper based system was that additional staff and resources were required to be able to cope with the added strain. Whilst there was some delay in making additional staff and resources available, staff acknowledged that when this was in place, it greatly alleviated pressure on wards. Additionally, the team ethos and willingness of staff from across the organisation to undertake administrative duties and work additional hours was considered one of the most positive lessons learned.

### What were the implications of the IT breakdown for patient safety?

A formal internal review, which consisted of a review of patient safety incidents reported on the hospital’s electronic Datix system was conducted by the hospital’s governance and risk team and concluded that no harm resulted from the outage. The majority of participants reported that they were not aware of any patient safety incidents during the outage and considered patient flow and delays to treatment and discharge to have been the main areas of concern. Whilst there was largely no perception of any ‘actual’ impact on safety, a number of participants from medical wards felt that, particularly at the beginning of the outage, that there was a potential for a major incident to occur, with this prevented only by good luck and staff working additional hours. Despite, the hospital’s internal review concluding that there were no patient safety incidents reported during the outage, junior doctors provided examples of situations where they felt patient safety had been, or could have been, at risk. One example is that of timeliness relating to microbiology results in order to know if an infection is resistant to the antibiotic the patient has been prescribed. When the IT system was down, these checks did not always happen and a consultant pathologist describes a situation which subsequently occurred:

*After that sort of chaos we had an untoward incident as well where…because normally we would rely on IT systems to do a lot of integrity checks on the samples…But unfortunately because it was just so chaotic and busy…the patient was treated unnecessarily…luckily the patient didn’t come to any harm but there could have been a serious untoward incident (Consultant pathologist)*

In comparison to their medical counterparts, surgeons perceived there to be a minimal risk to patient safety during the outage due to any potential issues being either pre-empted or worked-around. This was attributed largely to guidance that was developed by the hospital board and senior clinical staff at the start of the outage, which was then communicated to all staff. The guidance enabled staff, through a flow chart, to categorise cases into those that could proceed and those that should be cancelled. Elective surgeries and operations requiring blood transfusion were cancelled unless they were life threatening. Priority was given to emergency cases, cancer patients and cases that had previously been cancelled.

*There weren’t patient safety issues, because we pre-empted them, so the issue with blood in particular we would not have started a case that theoretically required transfusion.” (Consultant surgeon)*

In addition to formal guidance, staff developed their own workarounds and methods for risk assessing situations, which where appropriate, enabled them to conduct operations safely. For example, operative lists were re-organised, so that the patients, which did not require blood products were conducted first. Operations that required blood or blood products were then scheduled for later in the day, to enable time for blood to be sought. For emergency cases, or comorbid patients, other tests (such as blood gas machines), or where possible previous test results were used as proxies for key information to enable operations to proceed. Staff also highlighted the importance of communication and explained that for surgical staff safety was, as usual, central to all discussions when assessing the feasibility of cases.

*“I think we’d have found workarounds, so for instance, if I’d have needed to do a complex major urgently and need that renal function, there would have been a blood analyser in the hospital somewhere that would have been able to give us enough basic information for us to probably proceed.” (Consultant surgeon).*

Surgical staff considered decision making surrounding operations to be ‘easy’ as operations requiring blood products would not proceed without blood being available. However, for one surgeon the main difficulties surrounded cases where it was unusual for blood to be required, but there was a ‘theoretical risk of haemorrhage.’ A number of participants also raised concerns regarding the time taken to access blood products during the outage, particularly for acute cases, where it is difficult to predict when blood will be required and delaying treatment can have negative consequences for the patients. Limited access to microbiology also was perceived to have negative implications on safety. For instance, MRSA tests are required prior to orthopaedic operations – delays to microbiology services during the outage therefore meant that risk assessment procedures were developed to ensure the safety of orthopaedic operations. An additional area of concern for surgical staff included the impact of cancelling elective patients on their caseload and hospital targets.

Many participants discussed the impact which the outage had on clinical decision-making and its relationship to patient safety. Opinion was often strong but divided. Consultants were largely indifferent towards having no access to routine blood tests during the crisis and perceived this to have had limited impact on their ability to manage patients and make clinical decisions. Whilst consultants acknowledged that having no access to routine blood tests was associated with ‘the odd risk’, this was considered to be counterbalanced by the fact that clinicians should not be reliant on blood results and should be able to use their clinical judgement, the patient’s history and other clinical tests when making decisions and managing patients. A number of consultants attributed their indifference to the fact that whilst training as junior doctors ‘large batteries of tests’ were not available and so the outage, has required them to go back to using ‘old fashioned clinical skills.’ Comparisons were also made to the Junior Doctors’ strike, during which a number of consultants felt a more streamlined service and more prompt decisions were made, despite less resource being available. However, it was acknowledged that as consultants they have the authority to say that certain investigations are not required and they are more confident at ‘sniffing out trouble.’ When discussing the over reliance of clinicians and in particular junior doctors on ordering batteries of tests, consultants raised concerns that medicine has recently become protocol driven and encourages over-investigation. This was perceived to have caused medicine to become overcautious with doctors concerned about the prospect of being blamed if a patient came to harm and all available tests had not been ordered.

*There was clearly pandemonium over wherever it occurred, but my initial reaction was, ah, well we’ll just have to go back to a bit of clinical intuition and common sense, rather than doing the serum rhubarb every day on 5,000 different patients, we might have to start using our brains and judgement. (Consultant surgeon)*

Contrastingly, junior doctors discussed how not all consultants were confident in making decisions without routine blood tests being available, with some consultants considered much more risk averse than others. Junior doctors and the health care assistant also emphasised the importance of having access to blood results for clinical decision making and patient management and provided a number of examples to illustrate this.

*A lot of our patients will have abnormal blood tests that can be normal for them. There were some people that were for example, receiving intravenous fluids because they have a normal renal function, we have no way of knowing whether that was their normal or whether that was a new thing or not. So I think people quite possible got treatment that they didn’t necessarily require because we tried to be as safe as possible, because we didn’t have that information from the trends. (Junior doctor)*

### What were the implications of the IT breakdown for patient experience?

A number of participants discussed how when a hospital is in crisis, the numbers of patients attending the hospital does not reduce and so inevitably, there was a perceived impact on patient flow and experience. There was a perceived slower throughput of patients through ED and increased time to discharge due to the added time it took to process results manually.

*There was no seating area, it was awful, and people were sat on the floor just awaiting blood results, and just for the patient flow as well, it hindered it as it came to a standstill, not just here but on the downstream wards and people in A&E so you’ve got your 4 hour targets so generally this is something that you would get back in that target period but obviously due to the delay it had a knock on effect so you were getting unnecessary admissions to the wards because of breach times, because you didn’t have the results.* (Health care assistant and patient flow manager)

The impact of the outage on patient flow, and consequently on patient experience, was considered a particular problem for staff working in ambulatory care. This was attributed to patients being streamed inappropriately from ED to ambulatory care to increase flow and avoid breaching ED waiting times, but also to the added time taken to manually process blood results. Given that waiting times for blood tests were between 6-8 hours, managing patient frustration was vital and so staff in the ambulatory care unit established a number of workarounds to improve patient flow and experience during the outage. Staff ensured that patients were kept informed of waiting times, encouraged patients to make any child care or transport arrangements necessary and where appropriate, offered patients alternatives to waiting on wards. For example, staff offered to call patients when they received their results, providing them with the opportunity to go home or wait in the hospital’s café as opposed to on the ward. The ambulatory care unit also stopped accepting GP admissions at 4 pm during the crisis as staff knew there would be insufficient time to process the patient’s results. These patients were, where clinically appropriate advised to return the next day.

*As soon as they came in on the admission, we made all our staff members say “all our systems are down, normally you would wait up to 2 hours, it can be up to a 6-8 hour wait” so if you have any children you need to plan for, picking them up etc. (Ambulatory care unit Nurse)*

An additional problem for patient experience related to situations where blood tests were repeated for the same patient. For example, as blood tests requested by the GP were not prioritised, a proportion of these may have exceeded the length of time for which they can be stored before the test would have to be repeated and the patient recalled.

## Discussion

Our qualitative study uncovered the implications for patient safety and experience that arose from the catastrophic outage of a hospital’s pathology IT system. Whilst there was an overriding perception that no actual harm occurred to patients, there was divided opinion about the potential for a major safety incident to occur. Formal guidance existed to assist staff to navigate the outage but there was predominantly a reliance on informal workarounds. Junior clinicians seemed to struggle without access to routine blood test results whilst senior clinicians seemed largely unperturbed. The outage saw patient experience being negatively affected with an extensive wait time for manually processed diagnostic tests, and the associated logistical issues for patients that accompanied this.

Despite large parts of Western healthcare systems being dependent on IT for their delivery, very little is known about how healthcare organisations respond to, or are affected by, major IT failures. The majority of existing evidence surrounding e-health focusses on the perceived or potential benefits of digitised healthcare.^1,3–18^ Given the lack of literature about IT failures, perhaps the only parallel literature relates to emergency preparedness and response to crisis situations. A pertinent example is that of a Norwegian research team who explored the determinants for the success of a single EDs response to a terrorist attack.^21^ The authors conclude that preparedness, competence and crisis management built on empowerment enables healthcare workers to trust themselves and each other to make professional decisions and creative improvisations in an unpredictable situation. This to some extent corresponds with the findings of our study, where participants attributed the organisations initial underestimation of the outage’s significance and lack of agreed hospital-wide crisis management plan to have resulted in perceived negative implications to patient safety and experience.^21^

The incident which this paper is based on could be viewed as isolated. However, unfortunate but large scale attacks on IT systems across the world have in recent years seen the NHS become a prime target. In May 2017, a worldwide cyber-attack dubbed WannaCry targeted Microsoft Windows operating systems and the NHS was one of its main victims. During the attack, a third of NHS trusts were affected with around 19,000 appointments and 6912 operations cancelled.^29^ In 2018, the UK Department of Health commissioned an independent report on the scale of the into the Wanacry cyber-attack and found that large scale IT vulnerabilities were and are still resident across most of the NHS estate.^29^ Whilst, the WannaCry cyber-attack is aetiologically different to the pathology IT outage we describe in this paper it is analogous, not in scale but in the disruption and impact that it had on patients. Additionally, the Wanacry cyber-attack has led to a growing awareness of the dependence of healthcare on IT and technology failures being high on the English NHS policy agenda.

As IT becomes more central to healthcare it is necessary for the (potential) risks to patient safety to be made explicit so that lessons can be learned from impromptu organisational responses to unplanned IT catastrophes. This includes an examination of the factors which affected an organisations ability to respond in this time of crisis. To this end, hospitals may wish to look to the safety management literature (Hollnagel et al., 2015).^31^ In our case study, it could be argued that the organisation’s response was focussed around a ‘Safety 1’ approach where it is presumed that things go wrong because of detectible failures with management approaches based on identifying the causes and contributory factors of these failures. In our study, the organisation’s crisis management approach was largely based around identifying the problem ‘the outage’ and trying to mitigate any adverse effects it may have through centralised methods of crisis management such as hospital-wide communications and ad-hoc crisis management plans. There has however been a relatively recent shift within the resilience literature, which calls for organisations to adopt a Safety II approach and a resilient healthcare view (Hollnagel et al., 2015).^31^ This approach recognises that given the inherent complexity of healthcare systems, treating them as either ‘functioning or not’ as in a Safety I approach is unrealistic and that in reality healthcare systems work safely by individuals making various adjustments and adaptations in order to match current conditions. Applying this to crisis management, requires organisations to explore, during and not only retrospectively after a crisis has happened, what is going right, how things work and to manage and foster performance variability (Braithwaite et al., 2015).^32^ For example, in our study we found examples of how staff and wards had adapted to new ways of working during the outage, albeit to varying degrees of success. Adopting a Safety II approach would require organisations to explore these in more detail and focus their response around these positive adaptations.

We also propose the following, more specific recommendations for hospitals to consider when preparing for potential technology downtimes that are based on our study’s findings (Table 2).

**Table 2.** Recommendations.

Recommendations	
Role-play	Traditionally, informatics departments have focussed on ensuring that IT systems are fully functional and available on a 24-7 basis. However, failures and ‘cyber-attacks’ are inevitable and so there is a need to ensure that hospitals are properly prepared, to ensure they are able to respond effectively. To achieve this, services will need to be deliberately ‘taken down’ to allow hospitals to test the robustness and adequacy of any back-up systems and crisis management plans.
Guidance and agreed workarounds	During the outage, staff working in areas where clear guidance and plans had been implemented and communicated to all staff (e.g. surgical wards) were of the opinion that there was a minimal risk to safety. To minimise risk and ensure staff perceive the environment in which they are working to be safe, clinical and managerial staff should work together to develop strategies for ensuring patient safety is not compromised. This may include: agreed workarounds (e.g. use of proxy tests to indicate where procedures can go ahead), developing decision trees and guidance for prioritising certain patients and procedures and holding regular meetings and/or safety huddles with relevant clinical and managerial staff.
Communication and engagement of clinical staff	Clear communication and involvement of all relevant stakeholders when responding to a crisis is key to ensuring that the potential scale of the problem is understood and management plans are implemented as intended. Clear communication between staff and patients is also important for optimising patient experience. In our study participants discussed how being transparent with patients from the outset about the outage and its potential impact on their care was essential to patient management. Adopting a flexible approach to patient management and offering alternatives, such as waiting at home rather than on wards, may help to reduce potential negative impacts on patient flow and patient experience.

### Implications for further research

The digitisation of healthcare services will undoubtedly bring benefits. However, it is important to understand the potential risks of digitising health services and in particular the impact of downtimes on patient safety and experience. Future research will need to utilise a range of observational and qualitative methods to address this evidence gap. More specifically, qualitative research exploring the potential risks of digitising health services and ways to mitigate against any negative impacts on patient safety and experience is recommended. Quantitative research to determine the impact of downtimes on key safety and performance indicators, would be of benefit, with quasi-experimental designs considered the optimum method of achieving this.

### Strengths and limitations

This study adds to a limited evidence base that has reported on how healthcare organisations respond to crisis, particularly catastrophic IT failures. The purposive sampling frame ensured that despite the challenges of recruiting participants to qualitative interviews during times of crisis a comprehensive range of views on the impact and organisation’s response to the outage are represented. The study’s main limitation is that as a case study, the findings represent the views of clinical and non-clinical staff from across a single large inner-city NHS hospital.

We chose not to include the patient perspective. This was a deliberate decision as many patients were unaware of the outage, and with the hospitals crisis management aimed at containing the outage, we did not want to raise its profile and increase patient anxiety by promoting our study. Our data was collected from November 2016 to February 2017 and so its current relevance may be questioned. However, given the limited evidence base that exists on crisis management and the pressure on healthcare organisations to become digitised globally it is likely that some of the lessons and experiences reported here will be of use to other healthcare organisations, preparing for or experiencing catastrophic IT failures. Equally, some of the lessons here (i.e. robust crisis management plans, and preparedness) are likely to be transferable to other crisis that are not related to technology failures. This is demonstrated in the applicability of the study’s findings to the work of Brandrud et al. (2017).^21^

## Conclusion

This study identified that catastrophic IT failures are associated with a perceived elevated risk to patient safety and negative impacts on patient experience. The potential risks of digitising healthcare is an under-researched area. Future mixed methods research should be prioritised to quantify the potential risks of digitising healthcare and identify ways to mitigate against this. This research also provides valuable lessons which may influence how hospitals prepare for unexpected technology downtimes. Given the international pressure on hospitals to become digitised, these lessons are particularly relevant.

## Supplemental Material

sj-pdf-1-dhj-10.1177_20552076211010033 - Supplemental material for What are the implications for patient safety and experience of a major healthcare IT breakdown? A qualitative study Click here for additional data file. Supplemental material, sj-pdf-1-dhj-10.1177_20552076211010033 for What are the implications for patient safety and experience of a major healthcare IT breakdown? A qualitative study by Arabella Scantlebury, L Sheard, Cindy Fedell and J Wright in Digital Health

sj-pdf-2-dhj-10.1177_20552076211010033 - Supplemental material for What are the implications for patient safety and experience of a major healthcare IT breakdown? A qualitative study Click here for additional data file. Supplemental material, sj-pdf-2-dhj-10.1177_20552076211010033 for What are the implications for patient safety and experience of a major healthcare IT breakdown? A qualitative study by Arabella Scantlebury, L Sheard, Cindy Fedell and J Wright in Digital Health
