# Supplementary material for: What are the implications for patient safety and experience of a major healthcare IT breakdown? A qualitative study
Source: Digit Health. 2021 Apr 19;7:20552076211010033. doi: 10.1177/20552076211010033 (PMC8060737; doi:10.1177/20552076211010033)
Supplement: sj-pdf-1-dhj-10.1177_20552076211010033 - Supplemental material for What are the implications for patient safety and experience of a major healthcare IT breakdown? A qualitative study [file sj-pdf-1-dhj-10.1177_20552076211010033.pdf]

Evaluating the impact of a pathology system power outage

UNIVERSITY of York

Topic guide

At the beginning of all interviews:

- Introduce self
- Introduce study
- About audio recording/anonymity/confidentiality
- Explain how data will be used
- Obtain Verbal and written consent
- Provide participant with the opportunity to ask questions

Main interview

- Job role, years experience
- What is the pathology system used for in routine practice.

- What is your understanding as to why the system 'crashed'?
- What was the impact of the system 'crashing'?
    - Impact on safety
    - Clinical practice
    - Impact on other trusts/other services e.g. communication with GP
    - Impact on the trust as a whole
    - Which areas of the trust do you think have been most affected?
- What are the long term impacts of the system crashing?
    - Is it still impacting on practice now?
- Were patients aware that the system has crashed? If so what was their reaction?
- How did the trust respond to the situation?
    - Communication,
    - back-up procedure
- What systems have been put in place to prevent this happening with other IT systems in the trust?

- Do you think something similar could happen again?
    - If so what would you do prevent this?
    - What do you think are the key lessons that have been learned?
    - Has there been anything positive about this experience?
        - Unintended consequences

End the interview:

- Thank participant ask if they have any other comments
- Explain again about how data will be used and reiterate about anonymity and confidentiality
- Provide opportunity for questions and states that the lead researcher is contactable after the interview should questions arise.
