# Supplementary material for: What are the implications for patient safety and experience of a major healthcare IT breakdown? A qualitative study
Source: Digit Health. 2021 Apr 19;7:20552076211010033. doi: 10.1177/20552076211010033 (PMC8060737; doi:10.1177/20552076211010033)
Supplement: sj-pdf-2-dhj-10.1177_20552076211010033 - Supplemental material for What are the implications for patient safety and experience of a major healthcare IT breakdown? A qualitative study [file sj-pdf-2-dhj-10.1177_20552076211010033.pdf]

Evaluating the impact of a pathology system power outage

- Introduce self and study
- Discuss anonymity, audio-recording, confidentiality**,** how data will be used
- Obtain consent
- Explain how focus groups work; may call on individuals, try to avoid talking over one another because of Dictaphone
- Opportunity to ask questions

Three questions

1. What was the impact of the system crashing?
   - **a.** On safety
   - **b.** Practice generally
   - **c.** Efficiency
   - **d.** On the trust as a whole
   - **e.** On other services e.g. communication with GPS
   - **f.** Were patients aware of the system crashing

2. How did the trust respond to the situation?
   - **a.** How were you informed/communication
   - **b.** Back up procedures

3. Was there anything positive that can be taken from the experience/lessons learned?
